# A Plant-Produced *in vivo* deglycosylated full-length Pfs48/45 as a Transmission-Blocking Vaccine Candidate against malaria

**DOI:** 10.1038/s41598-019-46375-6

**Published:** 2019-07-08

**Authors:** Tarlan Mamedov, Kader Cicek, Kazutoyo Miura, Burcu Gulec, Ersin Akinci, Gunay Mammadova, Gulnara Hasanova

**Affiliations:** 1 Akdeniz University, Department of Agricultural Biotechnology, Dumlupınar Boulevard 07058 Campus, Antalya, Turkey; 2 Azerbaijan National Academy of Science, Department of Biology and Medical Science, 24 Istiglaliyyat Street, Baku, Azerbaijan; 3 Laboratory of Malaria and Vector Research, National Institute of Allergy and Infectious Diseases, National Institutes of Health, 12735 Twinbrook Parkway, Rockville, MD USA

**Keywords:** Biotechnology, Vaccines

## Abstract

Pfs48/45 is a leading antigen candidate for a transmission blocking (TB) vaccine. However, efforts to produce affordable, safe and correctly folded full-length Pfs48/45 using different protein expression systems have not produced an antigen with satisfactory TB activity. Pfs48/45 has 16 cysteines involved in disulfide bond formation, and the correct formation is critical for proper folding and induction of TB antibodies. Moreover, Pfs48⁄45 is not a glycoprotein in the native hosts, but contains potential glycosylation sites, which are aberrantly glycosylated during expression in eukaryotic systems. Here, we demonstrate for the first time that full length, Endo H *in vivo* enzymatic deglycosylated Pfs48/45 antigen is produced at a high level in plants and is structurally stable at elevated temperatures. Sera from mice immunized with this antigen showed strong inhibition in SMFA. Thus, Endo H *in vivo* enzymatic deglycosylated Pfs48/45 is a promising candidate for the development of an affordable TB vaccine, which may have the potential to save millions.

## Introduction

Malaria is a common infectious disease caused by protozoan parasites of the genus *Plasmodium* and transmitted by the female Anopheles mosquito. According to the latest World Malaria Report^1^, there were 219 million cases of malaria in 2017 with 435 000 deaths and nearly half of the world’s population was at risk of malaria. Although there have been many decades of effort, malaria remains the leading cause of morbidity and mortality among the human population globally. *Plasmodium falciparum* is one of the five species of malaria parasites and is responsible for the majority of deaths. Although several vaccines are currently under development, no vaccine is currently available that provides a satisfactory level of protection against malaria. Pfs48/45 is one of the leading candidates for transmission blocking (TB) vaccine development^2^ and has been shown to play an essential role in parasite fertilization. The production of correctly folded recombinant Pfs48/45 has been the main obstacle to the development of a Pfs48/45-based vaccine. Pfs48/45 is a complex protein and has seven potential N-linked glycosylation sites and contains 16 cysteines that are involved in disulfide bond formation. Correct formation of disulfide bridges is essential for proper folding of cycteine-rich proteins. The Pfs48/45 protein is synthesized exclusively during gametogenesis, present on the surface of gametes and zygotes^3^, but absent in asexual stages^4,5^. According to a number of reports^6–9^ Plasmodium has the potential to have O and N-glycans, however; it has been demonstrated that the Pfs48⁄45 protein does not possess N-linked glycans^10,11^. On SDS-PAGE, under non-reducing conditions the native Pfs48/45 molecule appears as a doublet protein with a molecular mass (MM) of 48 and 45 kDa, probably due to two different disulfide confirmations, but it shows a single 58 kDa band under reducing conditions^3^. Expression of Pfs48/45 has been attempted using several systems such as Baculovirus in Sf9 cells (Full-length Pfs48/45)^5^, *Escherichia coli* (GST-Pfs48/45, aa 56–401)^10^, Vaccinia Virus (Full-length Pfs48/45)^10^, *Saccharomyces cerevisiae* (Full-length Pfs48/45)^12^, *Pichia pastoris* (Full-length Pfs48/45)^12^, *E*. *coli* (cytosole) (CH-rPfs48/45)^13^ (Full-length Pfs48/45)^14^, *E*. *coli* (Periplasm) (MBP-10C)^15^ *Chlamydomonas reinhardtii* (Pfs48/45, aa 178–448)^16^, HEK293 (Pfs48/45−NGln and Pfs48/45+NGln)^17^, *Lactococcus lactis* (R0.6C, aa 291–428)^18^, (R0.10C aa 159–428)^2,19,20^. However, efficient and conformationally-correct expression of full-length Pfs48/45 was problematic (no or low expression, poor solubility, did not elicit TB antibodies in Balb/c mice, etc.) in most used expression platforms^10,12^. No or low expression level of full length Pfs48/45 protein was observed in *S*. *cerevisiae* and *P*. *pastoris*, respectively, probably due to hyper-mannosylation^12^. Pfs48/45 protein containing 10 cysteine residues (a C-terminal fragment) was expressed in *E*. *coli* as a fusion protein, which was shown to be correctly folded and elicited functional transmission blocking antibodies in mice^15^. However, the full-length form of Pfs48/45 did not properly fold^15^ and had poor solubility, a low expression level, and much weaker epitope recognition^15^. Later, using a codon optimization strategy, the full-length Pfs48/45 was produced in *E*. *coli* periplasm, which showed 93% reducing activity in standard membrane-feeding assay (SMFA)^13^. A 6C domain has been expressed using different expression systems^18,21^. However, since a 6C-fragment is smaller in size containing epitope I only, but excluding epitopes IIb and III, a higher dose of 6C protein was needed to elicit optimal TRA by SMFA, compared with 10C or full length Pfs48/45^18^. It should be noted that, the crystal structure of high-potency transmission blocking antibody in complex with Pfs48/45 6C has been recently solved, which would be important for developing transmission blocking vaccines by improving the vaccine design^21,22^.

Numerous studies in recent years have demonstrated plant expression systems promising expression platforms for cost-effective, fast and safe production of a variety of recombinant proteins^23–26^. Plant expression systems have a number of advantages compared to other expression systems currently used, and have the ability to accumulate hundreds of milligrams of target protein per kilogram of biomass in less than a week. This system has been successfully used for rapid and cost-effective production of a variety of recombinant vaccine candidates^24,27–29^. However, the ability of the plant expression systems to glycosylate proteins limits this system for the production of some proteins, for example, variety of bacterial proteins and malaria antigens, which are important for pharmaceuticals. Native Pfs48/45 proteins of *P*. *falciparum* are complex proteins but do not carry N-linked glycans. These proteins contain potential N-linked glycosylation sites, therefore, are aberrantly glycosylated during expression in most of eukaryotic systems, including plants^11,24,29^. There were attempts to produce a full-length Pfs48/45 protein in *N*. *benthamiana* plant using a transient expression system, but the resulting plant produced antigen showed very low TB activity^11,30^. It was hypothesized that the low TB activity may be associated with aberrant N-glycosylation of Pfs48/45 in plant system. To address this issue, a robust strategy to produce target proteins of interest in the non-glycosylated form by co-expression of target proteins of interest with bacterial PNGase F^11^ has been developed. Using the PNGase F *in vivo* deglycosylation strategy, it was shown that the PNGase F deglycosylated form of Pfs48/45 was recognized by epitope-specific monoclonal antibodies against epitopes I, III, and V 2- to 6-fold better than the glycosylated form^11^. Notably, deglycosylation of proteins with PNGase F causes amino acid changes in produced target proteins, due to the deamidation of asparagine to aspartate in the N-X-S/T site^11,31^. A more advanced strategy for production of target proteins in eukaryotic systems without the concomitant deamidation of asparagine in amino acid sequences has recently been developed^24^. Using this approach, we recently demonstrated that unlike the glycosylated counterpart, the recognition of Endo H or PNGase F deglycosylated forms of Pfs48/45 by a conformational Pfs48/45-specific monoclonal antibody was similar. Since SMFA is a powerful and pivotal method to evaluate TB vaccine efficacy, the next step was to evaluate the different non-glycosylated variants of Pfs48/45 in SMFA analysis to select the best antigen target for further TB vaccine development. In the current study, non-glycosylated full length Pfs48/45 and Pfs48/45-10C of *P*. *falciparum* were produced in *N*. *benthamiana* plant, and the purified proteins were injected into mice to generate antisera for immunogenicity and SMFA analysis. We demonstrated that, IgGs from Endo H *in vivo* deglycosylated full length Pfs48/45 had strong inhibition in SMFA compared to PNGase F deglycosylated full length Pfs48/45, and also Endo H and PNGase F deglycosylated Pfs48/45-10C counterparts. However, under the same test conditions, IgGs from glycosylated Pfs48/45 variants displayed insignificant inhibition.

## Results

### Modifications in production and purification for Pfs48/45 recombinant proteins

**Figure 1:**
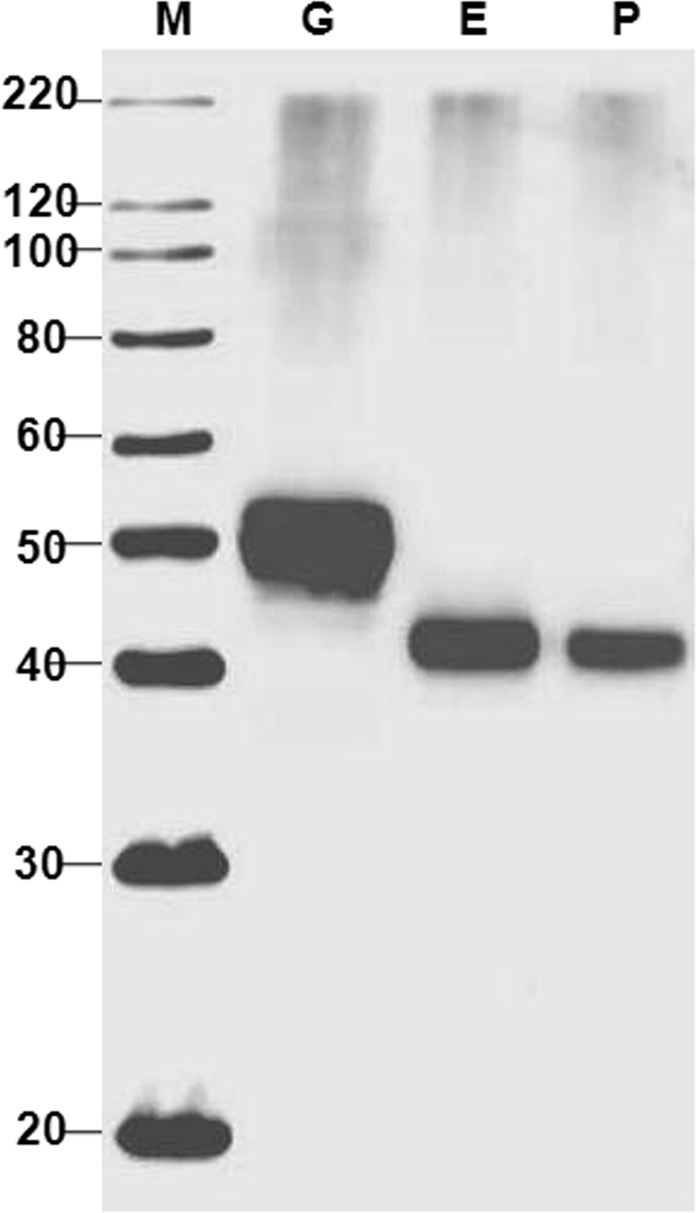
Western blot analysis of co-expression Pfs48/45-10C with bacterial Endo H or PNGase F in *N*. *benthamiana* plants. *N*. *benthamiana* plant was infiltrated with the pEQ-Pfs48/45-10C-FLAG-KDEL construct, for the production of glycosylated Pfs48/45-10C-FLAG (G). *N*. *benthamiana* plants were infiltrated with combinations of the pBI-Endo H/pEQ-Pfs48/45-10C-FLAG-KDEL or pBI-PNGase F/pEQ-Pfs48/45-10C-FLAG-KDEL constructs, for the production of Endo H (E) or PNGase F (P) deglycosylated Pfs48/45-10C-FLAG proteins. After centrifugation of the crude extract, samples were run on SDS–PAGE, followed by western blotting. Proteins on the blot were probed with anti-FLAG antibody. M: MagicMark XP Western Protein Standard. The image was taken using the highly sensitive GeneGnome XRQ Chemiluminescence imaging system.

**Figure 2:**
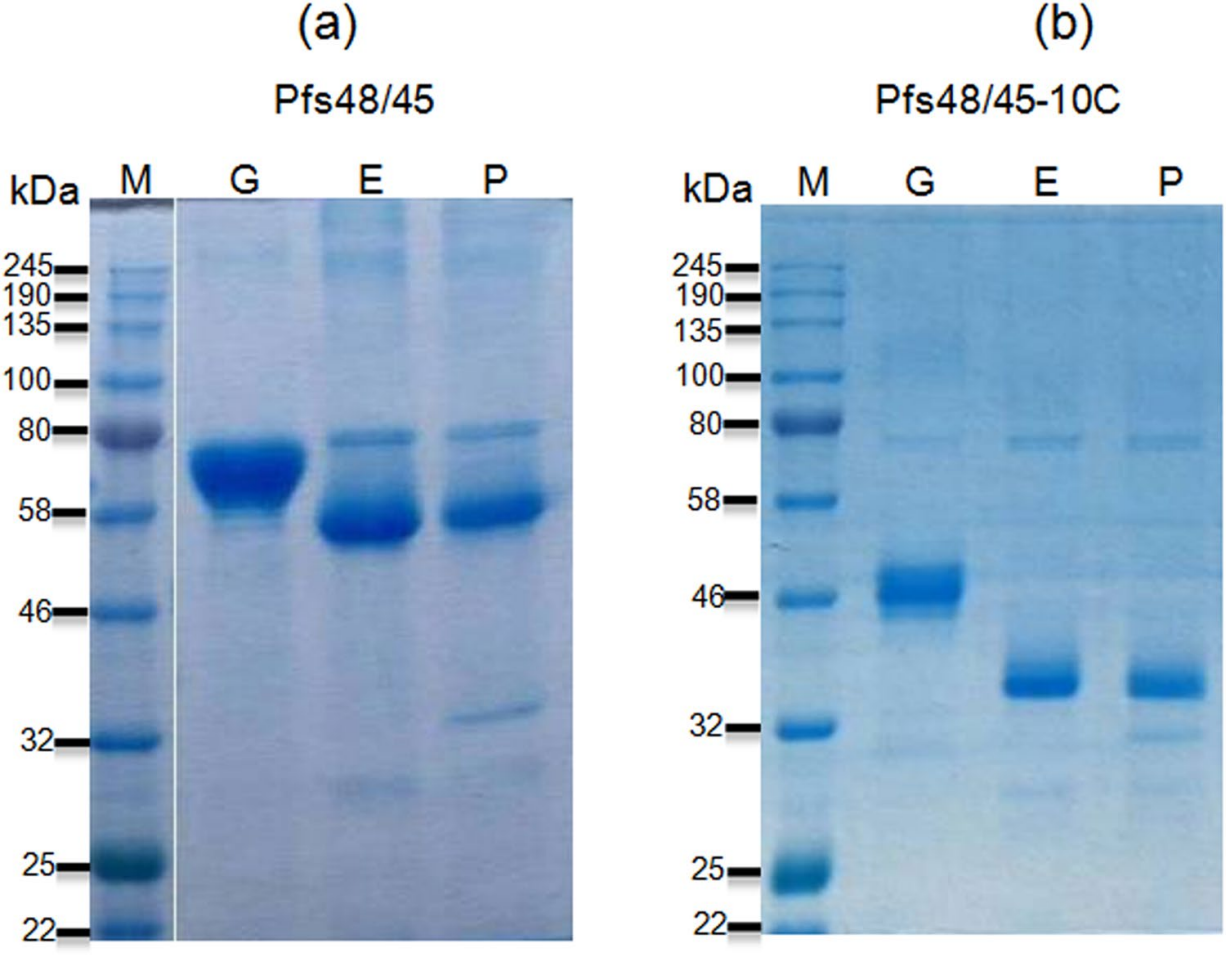
SDS-PAGE analysis of purified, plant produced Pfs48/45 and Pfs48/45-10C variants. (**a**) SDS-PAGE analysis of purified, plant produced full length Pfs48/45 variants; (**b**) SDS-PAGE analysis of purified, plant produced Pfs48/45-10C variants. Lanes were loaded with ~3.0 μg per lane for glycosylated (G), Endo H (E) or PNGase F (P) *in vivo* deglycosylated plant produced Pfs48/45 or Pfs48/45-10C proteins. M: color prestained protein standard.

Since the expression level and purification yield of His tagged deglycosylated Pfs48/45-10C proteins were low and in addition, aggregation of the PNGase F deglycosylated Pfs48/45-10C was observed^24^, in this study we therefore constructed a new plasmid, expressing FLAG tagged Pfs48/45-10C. Figure 1 demonstrates western blot analysis of FLAG tagged Pfs48/45-10C expressed alone (gPfs48/45-10C; Fig. 1, lane G) or co-expressed with deglycosylation enzymes Endo H (dPfs48/45 (E)-10C; Fig. 1, lane E) or PNGase F (dPfs48/45 (P)-10C; Fig. 1, lane P). The western blot analysis performed with crude extracts (plant harvested at 6 days post-infiltration,dpi) demonstrates a shift in the mobility of dPfs48/45 (E)-10C and dPfs48/45 (P)-10C compared to that of gPfs48/45-10C, indicating that FLAG tagged Pfs48/45-10C was enzymatically deglycosylated *in vivo*. Purifications of all 6 variants, full length Pfs48/45 and Pfs48/45-10C proteins were performed by anti-FLAG affinity chromatography using anti-DYKDDDDK affinity gel as described in Methods. Figure 2 demonstrates SDS-PAGE analysis of plant produced, affinity purified glycosylated or deglycosylated Pfs48/45 or Pfs48/45-10C proteins. To increase the yield of full length Pfs48/45 variants [gPfs48/45, dPfs48/45 (E), dPfs48/45 (P)], the purification method has been improved. Purity of the purified proteins were determined by SDS-PAGE, western blot, as well as by RP-HPLC. The purity of gPfs48/45, dPfs48/45 (E), dPfs48/45 (P) determined by SDS-PAGE and Western blot were ~90%, ~60% and ~50%, respectively. The purity of gPfs48/45, dPfs48/45 (E), dPfs48/45 (P) determined by RP-HPLC were ~89%, ~80% and ~65%, respectively. The purification yields of gPfs48/45, dPfs48/45 (E) and dPfs48/45 (P) were calculated as ~94, ~52 and ~27 mg/kg leaf biomass, respectively. In the previous study^24^ the purification yields were ~57, ~28, ~16, and about 1.6–1.7-fold improvement was observed. For the 10C variants, the purity of gPfs48/45-10C, dPfs48/45 (E)-10C, dPfs48/45 (P)-10C determined by SDS-PAGE and Western blot were ~80%, ~50% and ~50%, respectively. The purity of gPfs48/45-10C, dPfs48/45 (E)-10C, dPfs48/45 (P)-10C determined by RP-HPLC were ~59%, ~67% and ~62%, respectively. The purification yields were 83, 44 and 36 mg/kg leaf biomass. Although the purification yield of His tagged gPfs48/45-10C was comparable (~75 mg/kg) with the FLAG tagged version, the purification yield for His tagged dPfs48/45 (E)-10C was ~12.5 mg/kg, and the purification yield of His tagged dPfs48/45 (P)- was much lower, <2.5 mg/kg, due to aggregation^24^.

### Stability assessment of different variants of Pfs48/45 or Pfs48/45-10C

**Figure 3:**
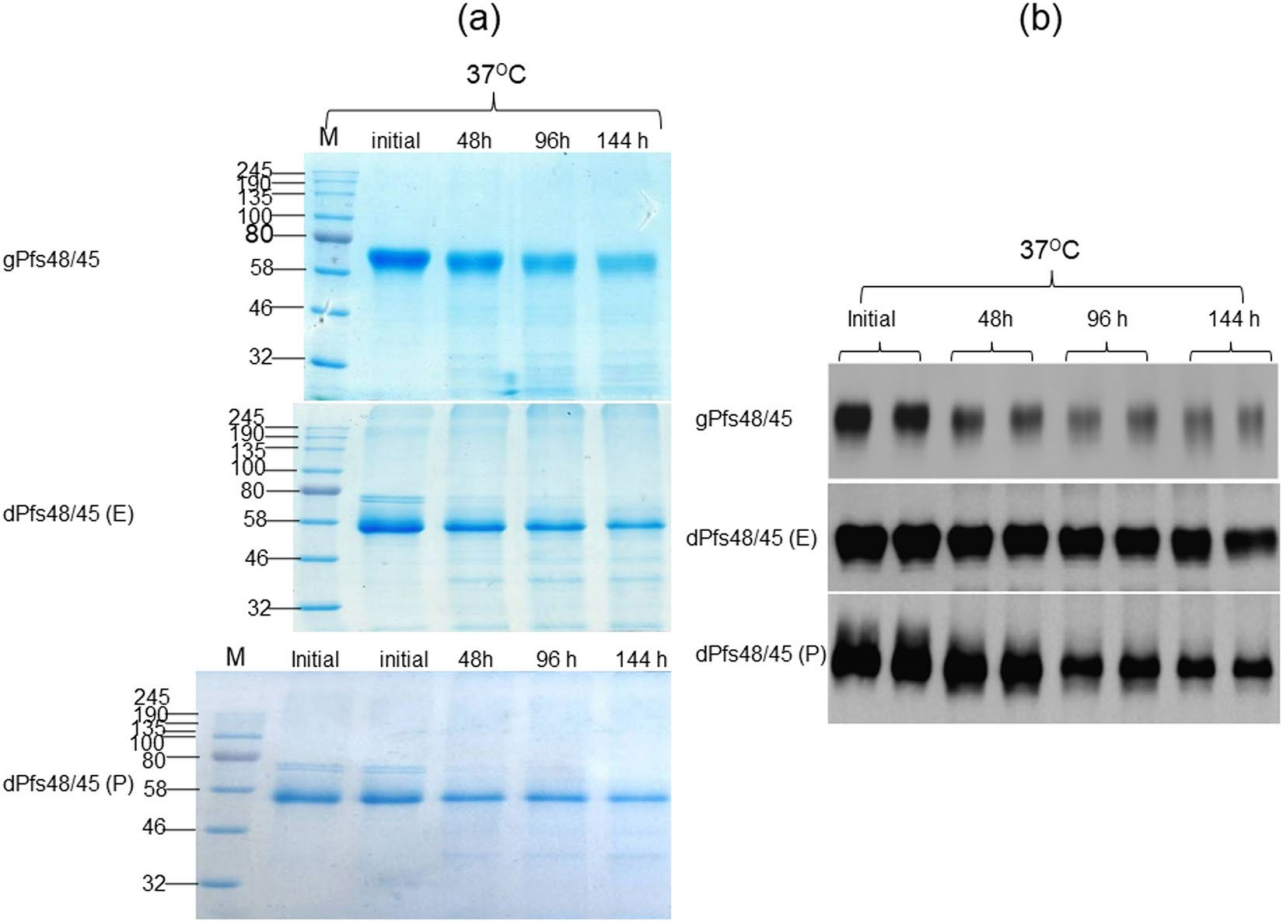
Stability of glycosylated and deglycosylated Pfs48/45 variants. Plant produced, FLAG antibody affinity column purified glycosylated Pfs48/45 (gPfs48/45) and *in vivo* Endo H (dPfs48/45 (E)) or PNGase F (dPfs48/45 (P)) deglycosylated variants of Pfs48/45 were incubated at 37 °C for 48, 96 and 144 hours, and analyzed in SDS-PAGE (**a**) or western blot (**b**). Lanes were loaded with ~2.0 μg per lane of Pfs48/45 proteins. M: color prestained protein standard. (**b**) Hundred ng per lane of Pfs48/45 proteins were run in SDS-PAGE prior to western blotting. Proteins on the blot were probed with anti-FLAG antibody. The image was taken using the highly sensitive GeneGnome XRQ Chemiluminescence imaging system.

**Figure 4:**
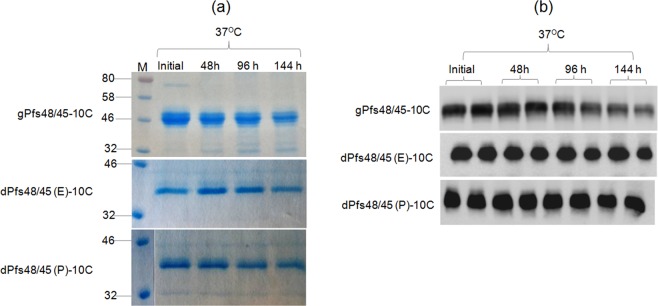
Stability of glycosylated and deglycosylated Pfs48/45-10C variants. (**a**) Plant produced, FLAG antibody affinity column purified glycosylated (gPfs48/45-10C) or *in vivo* Endo H (dPfs48/45 (E)-10C) or PNGase F (dPfs48/45 (P)-10C) deglycosylated forms of Pfs48/45-10C variants were incubated at 37 °C for 48, 96 and 144 hours, and analyzed in SDS-PAGE. Lanes were loaded with ~2.0 μg (gPfs48/45-10C) or ~1.5 μg (dPfs48/45 (E)-10C or dPfs48/45 (P)-10C) of protein. M: color prestained protein standard. (**b**) Hundred ng per lane of Pfs48/45-10C proteins were run in SDS-PAGE prior to western blotting. Proteins on the blot were probed with anti-FLAG antibody. The image was taken using the highly sensitive GeneGnome XRQ Chemiluminescence imaging system.

**Figure 5:**
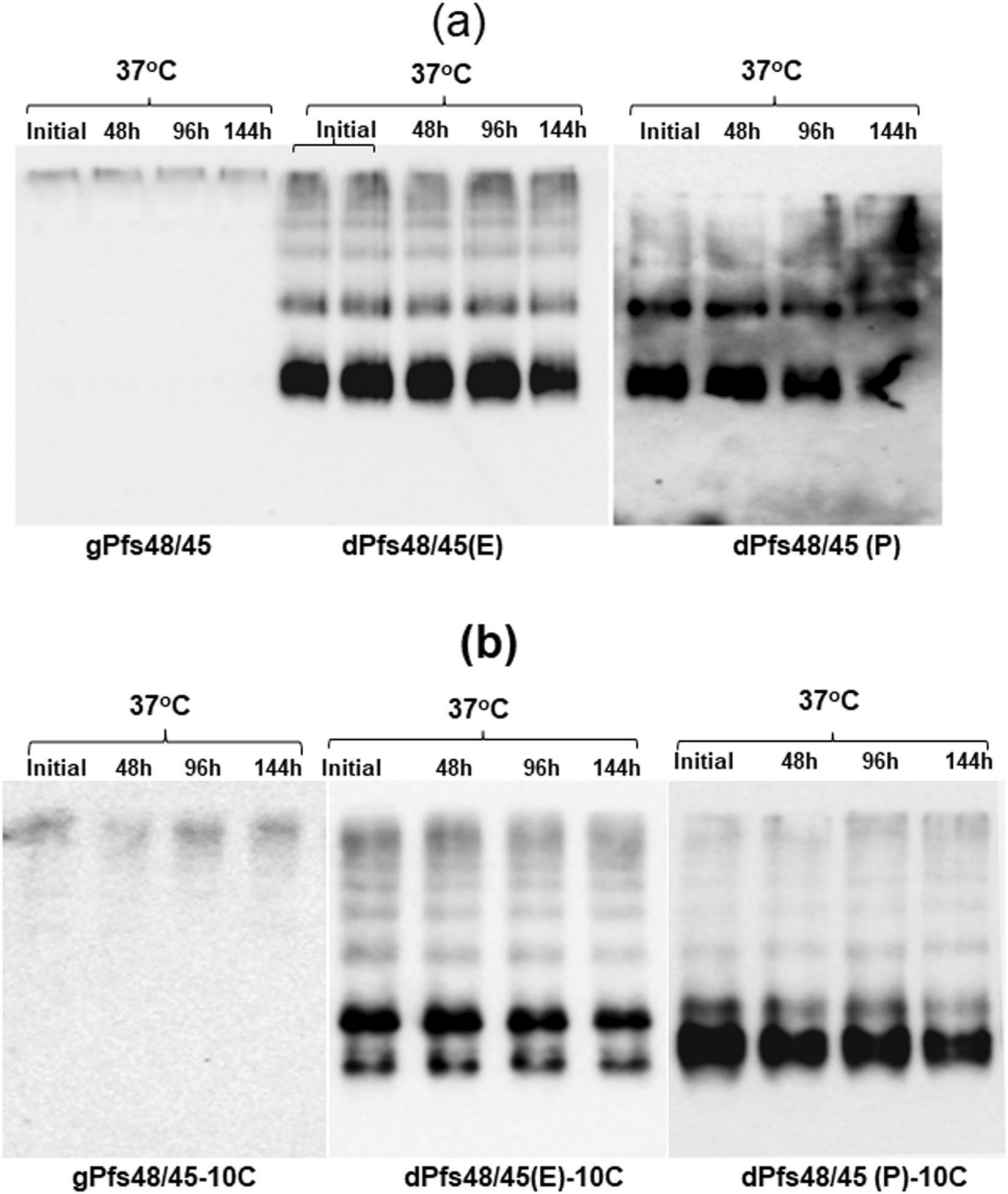
Stability analysis of glycosylated and deglycosylated Pfs48/45 and Pfs48/45-10C variants using conformational specific Pfs48/45 monoclonal antibody. Glycosylated and deglycosylated Pfs48/45 and Pfs48/45-10C variants were incubated at 37 °C for 48, 96 and 144 hours. Two hundred ng per lane of deglycosylated proteins, as indicated, were run in Native PAGE prior to western blotting. Proteins on the blot were probed with the conformational specific Pfs48/45 monoclonal antibody, IIC5B10-1 (MRA-316). (**a**) Glycosylated and deglycosylated Pfs48/45; (**b**) Glycosylated and deglycosylated Pfs48/45-10C.

**Figure 6:**
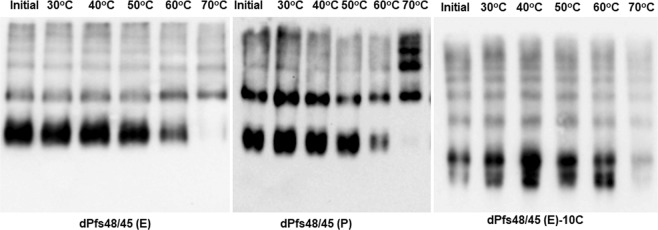
Effect of temperature on the recognition of deglycosylated Pfs48/45 proteins with conformational specific Pfs48/45 monoclonal antibody. Deglycosylated dPfs48/45 (E), dPfs48/45 (P) and dPfs48/45 (E)-10C proteins, as indicated, were incubated at different temperatures as indicated for 15 min and then proteins were run on Native PAGE followed western blotting. Proteins on the membrane were detected using a conformational specific Pfs48/45 monoclonal antibody, IIC5B10-1 (MRA-316).

The stability of plant produced glycosylated and *in vivo* deglycosylated forms of Pfs48/45 and Pfs48/45-10C were examined after incubation at 37 °C for a longer period of time: 48, 96, and 144 hours (Figs 3 and 4), using a method similar to those described previously^24^. For calculating the degradation of Pfs48/45 variants, protein bands of each sample from coomassie blue stained on SDS-PAGE and western blot were quantified using ImageJ software. These analyses showed that gPfs48/45 was about 60–65% degraded at 37 °C for 144 hours, however, at the same condition, degradations of dPfs48/45 (E) and dPfs48/45 (P) were no more than 30%. For 10C variants, gPfs48/45-10C protein was degraded about 40–50% at 37 °C for 144 hours, however, very little (less than 5%) degradation was observed for dPfs48/45 (E)-10C and dPfs48/45 (P)-10C. The stability of plant produced glycosylated and *in vivo* deglycosylated forms of Pfs48/45 and Pfs48/45-10C were also monitored by using a conformational antibody (Fig. 5). These results demonstrate that deglycosylated Pfs48/45 proteins are structurally stable at an elevated temperature for prolonged periods. We also performed western blot analysis of plant produced, deglycosylated Pfs48/45 and Pfs48/45-10C proteins incubated at different temperatures (30 °C, 40 °C, 50 °C, 60 °C and 70 °C) for 15 min, using a conformational specific Pfs48/45 antibody (Fig. 6). Based on these results it was confirmed that plant produced, deglycosylated Pfs48/45 and Pfs48/45-10C proteins were structurally stable and recognized by the conformational specific Pfs48/45 antibody when incubated at 50 °C for 15 min. All of the above experiments were examined 2–3 times with similar results.

### SMFA

**Figure 7:**
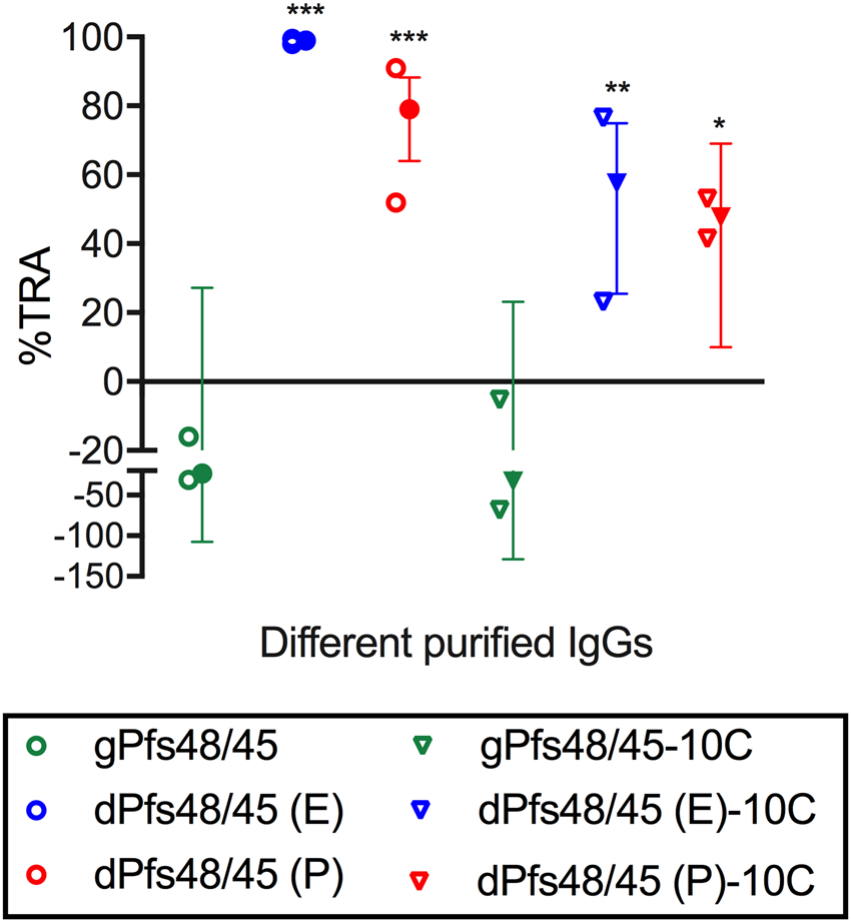
Transmission reducing activity of anti-Pfs48/45 IgGs. Purified total IgGs were tested at 0.75 mg/mL with complement in two independent assays. Individual feeding data (open symbols), and the best estimate (closed symbols) with 95% confidence intervals (bars) of % inhibition (%TRA) from the two feeds are shown. Significant inhibitions are indicated with asterisks: ***p < 0.001; **p < 0.01; *p < 0.05.

Immunization of mice with Pfs48/45 or Pfs48/45-10C antigens and the preparation of serum was performed as described in the Methods section. To evaluate the biological activity of induced antibodies, a total IgG was purified from a pool of antisera in each group, and the purified IgGs were tested at 0.75 mg/mL with complement in two independent SMFAs (Fig. 7 and Supplementary Table S1). Based on the two assays, all 4 deglycosylated groups (dPfs48/45 (E), dPfs48/45 (P), dPfs48/45 (E)-10C and dPfs48/45 (P)-10C) showed significant inhibitions, while IgGs from both glycosylated groups (gPfs48/45 and gPfs48/45-10C) tested under the same conditions did not display significant inhibition in SMFA. Among the four deglycosylated groups, dPfs48/45 (E) IgG showed the strongest activity. The results indicated that deglycosylation of the recombinant proteins was required to induce functional antibodies.

**Figure 8:**
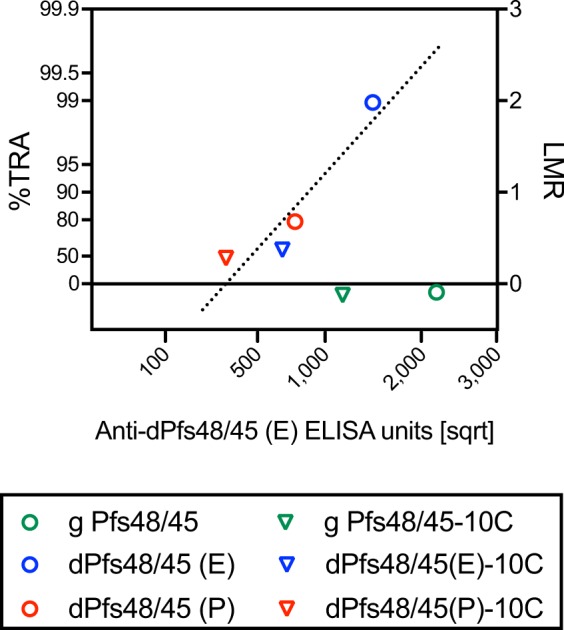
Correlation between ELISA units and SMFA activity. Anti-dPfs48/45 (E) ELISA units (on a square root scale, x-axis) and the associated the best estimated %TRA value from two feeds (on the left side of the y-axis) of each test IgG are shown. The ratio of mean oocyst (mean oocyst density in control divided by mean in test) is plotted on a log scale (LMR, right side of y-axis).

Next, the correlation between anti-dPfs48/45 (E) ELISA (enzyme-linked immunosorbent assay) units (EU) and SMFA activity was assessed. As shown in Fig. 8, the two glycosylated groups showed relatively higher anti-dPfs48/45 (E) EU among the 6 samples, yet did not inhibit oocyst formation in SMFA. For the other 4 groups, there was a strong linear correlation between EU and LMR (log-transformed mean oocyst ratio between test and control IgGs) (R^2^ = 0.87 by a linear regression), indicating that there was no difference in the quality of antibodies. In other words, the 4 IgGs would show the same level of SMFA activity at the same anti-dPfs48/45 (E) EU while the IgGs were elicited against 4 different recombinant proteins.

## Discussion

*P*. *falciparum* is one of the most deadly parasites in human history and therefore, the development of a safe, low-cost and highly efficient malaria vaccine with long-term stability is urgently needed. It has been shown that the *Pf*s48/45 antigen is one of the leading candidates for transmission blocking^2,32^. However, since the full length *Pf*s48/45 is a cysteine rich protein, and its proper folding depends on the correct formation of disulfide bridges, it has been challenging to achieve high yield production, stable, and functionally active full length *Pf*s48/45 using different expression systems. Full length Pfs48/45 antigens have been produced using different systems, but most of them did not induce TB sera in mice or were not recognized by transmission-blocking antibodies^10,12,15^. However, sera from mice immunized with codon optimized full-length Pfs48/45, which was produced in *E*. *coli* periplasm, showed 93% inhibition in SMFA, supporting the view that alternative expression platforms are needed to further improve the production level, efficacy, stability, safety and affordability of Pfs48/45 based malaria vaccine.

In this study, we demonstrate for the first time that the full length, Endo H *in vivo* enzymatic deglycosylated Pfs48/45 antigen is produced at a high level in *N*. *benthamiana* plants and is structurally stable at elevated temperatures for a prolonged period. Moreover, sera from mice immunized with this plant produced malaria antigen showed strong inhibition activity in SMFA. Notably, the plant transient expression system becomes one of the promising expression platforms for a variety of recombinant proteins, including complex proteins and has a number of advantages including rapid, high production capacity and high scalability^23,25,28,33–35^. In addition, the plant expression system has been shown to be a safe platform due to the lack of mammalian pathogens and low level contamination with endotoxins^23,34,36^. Using a plant transient expression system, in previous efforts, a full length Pfs48/45 protein was produced in *N*. *benthamiana* plant, but the TB activity of this antigen was very low^11^. We hypothesized that it could be due to aberrant N-glycosylation of the Pfs48/45 protein. To address this need, in our previous efforts, we developed a strategy for the production of proteins in a non-glycosylated form, by co-expression of target proteins with the bacterial deglycosylation enzyme, PNGase F^11,30^. It was demonstrated that though removal of oligosaccharides from glycosylated proteins by PNGase F does cause an amino acid change in the produced deglycosylated protein, the strategy significantly improved the protein quality^11,24,29^. Since another deglycosylation enzyme, bacterial Endo H that catalyzes cleavage between two acetylglucosamine (GlcNAc) residues of the chitobiose core of N**-**linked oligosaccharides with great efficiency without the concomitant deamidation of asparagine^37–39^, we further thought to develop a procedure to produce non-N-glycosylation recombinant proteins in a non-glycosylated form by co-expression of target proteins of interest with Endo H while preserving their native conformation. Using this strategy^24^, we have recently produced six variants of Pfs48/45 and of Pfs48/45-10 in *N*. *benthamiana* plant, and purified proteins were tested using the conformational specific Pfs48/45 monoclonal TB antibody. It was demonstrated that plant produced *in vivo* Endo H deglycosylated Pfs48/45 and Pfs48/45-10C antigens were recognized by the conformational specific Pfs48/45 monoclonal antibody, in a manner similar to its PNGase F deglycosylated counterpart. In the current study, we improved the purification yield of both full length Pfs48/45 and Pfs48/45-10C. In the previous study^24^, the full length, FLAG tagged Pfs48/45 variants were purified using an anti-FLAG antibody column chromatography. In this study, the purification procedure was optimized as described in the Methods section and resulted in ~ a 1.6–1.7 fold increase in the purification yield. In the previous study^24^ the expression level and purification yield of His_6_ tagged, deglycosylated Pfs48/45-10C proteins were low. Moreover, some level of aggregation of the PNGase F deglycosylated Pfs48/45-10C was observed^24^. Therefore, in this study, we constructed a new plasmid, expressing FLAG tagged Psf48/45-10C, which resulted in a significant increase in the purification yield of deglycosylated variants. As reported earlier^24^, the expression levels, solubility and purification yields of dPfs48/45 (E) and dPfs48/45 (E)-10C proteins were higher than those of dPfs48/45 (P) and dPfs48/45 (P)-10C counterparts. The yield of malaria antigens could be enhanced after optimization of the co-expression conditions in *N*. *benthamiana* plants. Thus, in this study we have generated six plant produced Pfs48/45 variants, including glycosylated, Endo H and PNGase deglycosylated full length Pfs48/45 and Pfs48/45-10C proteins and improved their purification methods, and purified proteins were used for comparative stability assessment, immunogenicity study, and SMFA analysis. Here, we demonstrated that both Endo H and PNGase F deglycosylated Pfs48/45 and Pfs48/45-10C proteins were structurally stable and showed resistance at elevated temperature for prolonged periods compared to glycosylated Pfs48/45 variants. This may significantly increase the life and duration of vaccine storage and reduce the cost significantly. These results are consistent with our recent studies on PA83 stability that show plant produced Endo H or PNGase F deglycosylated forms of PA83 appeared to be more stable compared to glycosylated counterparts at elevated temperatures. Notably, PA83 of *Bacillus anthracis*, one of the three proteins composing the anthrax toxin, contains six potential N-linked glycosylation sites, but is not glycosylated in its native host, and is aberrantly glycosylated when produced in most of eukaryotic expression systems including plants^11,24,29^.

Although the stability of plant produced Endo H and PNGase F deglycosylated Pfs48/45 and Pfs48/45-10C proteins were similar at 37 °C for 144 hours, the purification yield of Endo H deglycosylated Pfs48/45 or Pfs48/45-10C proteins were about two times higher than that of plant produced PNGase F counterparts. Moreover, sera from mice immunized with dPfs48/45 (E) showed the strongest inhibition in SMFA compared to the PNGase F deglycosylated counterpart, dPfs48/45 (P), or two variants of deglycosylated Pfs48/45-10C. Of note, under the same test conditions, IgGs from glycosylated Pfs48/45 or Pfs48/45-10C groups did not display significant inhibition. Overall all the above findings demonstrate that the plant produced, Endo H *in vivo* deglycosylated, full length Pfs48/45 malaria antigen is a promising candidate for the development of an affordable, safe, stable, and full length Pfs48/45-based transmission blocking malaria vaccine, and therefore may have the potential to save millions.

In summary, our results demonstrate that the expression level and purification yield of the dPfs48/45 (E) protein is high and the protein is highly stable at elevated temperatures for a prolonged time, and, importantly, has strong inhibition activity in SMFA. All of these features support further characterization, formulation, pre-clinical testing and finally early stage clinical development of plant produced Endo H deglycosylated full length dPfs48/45 (E).

## Methods

### Cloning and co-expression of Endo H or PNGase F with Pfs48/45 and Pfs48/45-10C

The sequences of Pfs48/45 (amino acids 28–428, GenBank accession number EU366251, the full-length form of Pfs48/45, 16 cysteine residues and seven putative glycosylation sites) was optimized for expression in *N*. *benthamiana* plants and was synthesized by GENEART AG (Thermo Fisher Scientific) as described previously^24^. Pfs48/45-10C (amino acids 159–428, GenBank accession number EU366251, truncated form of Pfs48/45, contains I, II, III epitops, 10 cycteine residues and five putative glycosylation sites)^10,15^ was amplified by PCR as a C-terminal FLAG tag Pfs48/45 gene, synthesized by GENEART AG, as a DNA template. Amplified PCR product was digested with *AgeI* and *Xho I* restriction enzymes and cloned into pEAQ vector^40^ to obtain pEAQ-Pfs48/45-10C-FLAG-KDEL. Plasmid construction, *Agrobacterium tumefaciens* transformation, plant infiltration and co-expression of Endo H or PNGase F (non-tagged versions, with KDEL but lacking the FLAG epitop) with Pfs48/45 and Pfs48/45-10C were performed as described previously^24^. To express FLAG tagged Pfs48/45-10C in *N*. *benthamiana* plant, pEAQ-Pfs48/45-10C-FLAG-KDEL plasmid was introduced into the Agrobacterium tumefaciens strain AGL1. AGL1 carrying the pEAQ-Pfs48/45-10C-FLAG-KDEL plasmid was then infiltrated into 6-7-week old *N*. *benthamiana* plants. To confirm the expression of FLAG tagged Pfs48/45-10C protein, leaf tissue was harvested at 6 dpi (day post infiltration) and homogenized in three volumes of extraction buffer (20 mM sodium phosphate, 150 mM sodium chloride, pH 7.4). After centrifugation of the crude extract at 13 000 g for 20 min, samples were run on SDS-PAGE, followed by transfer to Polyvinylidene fluoride (PVDF) membranes (Millipore, Billerica, MA) for western blot analysis. The FLAG tagged plant produced recombinant Ffs48/45-10C was detected using anti-FLAG mAb (Cat. No.637301, BioLegend), followed by HRP goat anti-rat IgG antibody (Cat. No. 405405, BioLegend). To co-express a FLAG tagged Pfs48/45-10C with Endo H, pEAQ-Pfs48/45-10C/pBI-Endo H (non-tagged) constructs were used for plant infiltration. Similarly, to co-express a FLAG tagged Pfs48/45-10C with PNGase F, pEAQ-Pfs48/45-10C/pBI-PNGase F (non-tagged) constructs were used for plant infiltration.

### Purification of Glycosylated and Deglycosylated forms of Pfs48/45 and Pfs48/45-10C

Purification of glycosylated and deglycosylated forms of full length Pfs48/45 variants from 25-30 g *N*. *benthamiana* plant leaves was performed as described previously^24^ with some modifications. In the previous work^24^, the purification of Pfs48/45 was performed from 10 g leaves as follows: after the plant debris was removed by filtration through Miracloth followed by centrifugation at 20,000 g for 25 minutes and then filtered through a 0.45 μm syringe filter (Millipore) the crude extract was passed through the anti-FLAG M2 affinity gel column once, and then, after washing, the proteins were eluted from the column using elution buffer and then concentrated. In this study, the purification procedure was optimized as follows: crude extract was mixed gently with equilibrated resin at room temperature or 4 °C for 5 hours using a platform shaker, and then the sample mixture was loaded onto a chromatography column, and the resin was allowed to settle and drain naturally, and then flow through the column multiple times to enhance the binding efficiency, before wash and elute.

Purification of glycosylated and deglycosylated forms of FLAG tagged Pfs48/45-10C proteins were purified from *N*. *benthamiana* leaves by using anti-FLAG antibody (BioLegend) column chromatography. For purification, 25–30 g of frozen leaves from each construct was ground in a binding buffer (1X PBS/0,5 M NaCl) using a mortar and a pestle. Plant debris was removed by filtration through Miracloth followed by centrifugation at 20,000 g for 10 min and then filtered through a 0.45 micron syringe filter. An anti-FLAG M2 affinity gel column (Anti-FLAG M2 Affinity gel, BioLegend) was prepared according to the manufacturer’s instructions. Thirty milliliters of a clear supernatant were loaded into 10 ml resin column equilibrated with binding buffer. A column was washed with 10 volumes of binding buffer. After washing the column, the bound proteins were eluted using 200 mM Glycine/150 mM NaCl pH 2.2 into tubes containing 1 M Tris solution to neutralize. The eluted fractions were analyzed on SDS-PAGE, combined and concentrated using Amicon Ultra 0.5 mL centrifugal filters. The concentrated samples were stored at −80 °C until use.

The purity of six plant produced Pfs48/45 variants was determined by SDS-PAGE, western blot and RP-HPLC analysis. Different amounts of BSA (1.0, 1.5, 2.0, 2.5, 3.0 and 4 ug) or plant produced FLAG tagged PNGase F protein (12.5, 25, 50 and 100 ng) were used as protein standards, in SDS-PAGE and western blot, respectively. Protein bands of each sample from SDS-PAGE and western blot were quantified by a highly sensitive Syngene gel imaging system using GeneTools Software and the ImageJ program.

### Stability assessments of different variants of Pfs48/45 or Pfs48/45-10C

Stability assessments of different variants of Pfs48/45 or Pfs48/45-10C were performed using similar procedure as described previously^24^. Plant produced glycosylated and deglycosylated variants of Pfs48/45 and Pfs48/45-10C were diluted to 0.5 mg/mL with PBS, and were aliquoted into polypropylene Eppendorf low-binding tubes. Proteins were then incubated at 37 °C for 48, 96 and 144 hours. After incubation, samples were analyzed by SDS-PAGE and western blotting. For SDS-PAGE analysis, the samples were mixed with SDS loading dye (5X) and stored at −20 °C until use. All samples were then run on SDS-PAGE and western blot for analysis. The degradation of Pfs48/45 and Pfs48/45-10C variants were quantified using highly sensitive Gene Tools software (Syngene Bioimaging, UK) and ImageJ software (https://imagej.nih.gov/ij) for Native PAGE samples, and running buffers were prepared without denaturants or SDS. Proteins separated by Native PAGE were probed using the anti-Pfs48/45 mAb (IIC5B10-1, MRA-316) antibody^41^ as described previously^24^.

### Effect of temperature on the recognition of plant produced, deglycosylated Pfs48/45 proteins with conformational specific Pfs48/45 antibody

Deglycosylated Pfs48/45 and Pfs48/45-10C proteins (0.2–0.5 mg/ml) were incubated at different temperatures (30 °C, 40 °C, 50 °C, 60 °C and 70 °C) for 15 min and then the proteins were run on Native PAGE followed western blotting. Proteins on the membrane were detected using a conformational specific Pfs48/45 monoclonal antibody^41^.

### SDS-PAGE and western blot analysis

SDS-PAGE and western blot analysis of plant produced Pfs48/45 and Pfs48/45-10C were performed as described previously^24^. The FLAG tagged Pfs48/45-10C and Pfs48/45 variants were detected using anti-FLAG mAbs (Cat. No. 637301, BioLegend) or the anti-Pfs48/45 mAb (MRA-316) antibody. Native PAGE of FLAG tagged Pfs48/45 or Pfs48/45-10C variants is performed using native samples and running buffers without denaturants or SDS as described previously^24^. The images of the protein bands were taken using the GeneSnap software on a GeneGnome and were quantified using the Gene Tools software (Syngene Bioimaging, UK).

### RP-HPLC

Reversed-phase High Performance Liquid Chromatography (RP-HPLC) analysis of plant produced Pfs48/45 and Pfs48/45-10C proteins (Supplementary Fig. S1) was performed using Agilent 1200 Series HPLC System (Agilent Technologies, Böblingen, Germany) with ODS3 Intersil column (250 mm × 4.6 mm × 5 μm) and water/acetonitrile (%100 Acetonitrile (A), Water (B) mobile phases). The injection volume was 10 µl, column temperature was 50 °C and total run with flow rate of 1 mL/min was 60 min. Gradient profile was as follows: 0. min 3% A, 97% B; 10.40. min 55% A, 45% B; 30. min 3% A, 97% B; hold 30 min. All peaks in the chromatogram was detected at 280 nm. The purity was calculated as a percentage of peak area in relation to total area of peaks.

### Animal studies

Animal experiments were carried out at Akdeniz University Experimental Animal Care Unit under permission of the Local Ethics Committee for Animal Experiments at Akdeniz University with the supervision of a veterinarian (Protocol number: 2014.03.04). The animal protocols were approved by the Local Ethics Committee for Animal Experiments at Akdeniz University, for the mouse studies. All animal experiments and methods were performed in accordance with the animal experimentation guidelines and regulations approved by the Local Ethics Committee for Animal Experiments at Akdeniz University.

A group of 5 BALB/c mice, 6–8 weeks were immunized intramuscularly (IM) on study days 0, 21 and 42 with 3 μg of Pfs48/45 or Pfs48/45-10C antigens (not total protein amounts) adsorbed to 2.0% Alhydrogel. After 3 weeks from the last injection, blood samples were drawn from the abdominal aortic artery of the mice and were centrifuged at 3000 rpm for 15 minutes at +4 °C to obtain serum. The resulting sera were kept at −80 °C for further use.

### Total IgG purification

For each group, a pool of antisera was generated, total IgG was purified using a protein G column (GE Healthcare, Pittsburgh, PA) according to the manufacturer’s instructions, and adjusted to a final concentration of 16 mg/ml in PBS.

### ELISA

The basic methodology of ELISA has been published elsewhere^42^. As the coating antigen, dPfs48/45 (E) was utilized for all purified IgG samples, regardless of immunogens.

### SMFA

The standardized methodology for performing the SMFA has been described previously^43^. Briefly, 16–18 day old gametocyte cultures of the *P*. *falciparum* NF54 line were mixed with test IgGs at 0.75 mg/ml. The final mixture was immediately fed to ~50 female *Anopheles stephensi* mosquitoes through a membrane-feeding apparatus. All feeding experiments were performed with human complement. Mosquitoes were kept for 8 days and were dissected (n = 20 per group for test IgGs, n = 40 for negative controls) to enumerate the oocysts in the midgut. Only midguts from mosquitoes with any eggs in their ovaries at the time of dissection were analyzed. The human serum and red blood cells used for the gametocyte cultures and feeding experiments were purchased from Interstate Blood Bank (Memphis, TN).

### Statistical analysis

The best estimate of % inhibition in oocyst density (% transmission reducing activity, %TRA), the 95% confidence intervals (95% CI), and p-values from either single or two feeds were calculated using a zero-inflated negative binomial random effects model (ZINB model) described previously^44^.

Our previous studies^45,46^ have shown that SMFA data can be approximated to a linear regression when the IgG concentration of a test sample in a feeder is plotted on a square root scale against the log-transformed mean oocyst ratio between test and control IgGs (LMR). Therefore, a linear regression was performed between anti-dPfs48/45 ELISA units (EU, square root scale) and LMR.

All statistical tests were performed in R (version 3.4.1) or Prism 7 (GraphPad Software, La Jolla, CA), and p-values < 0.05 were considered significant.

## Supplementary information

Supplementary Information
