# Supplementary material for: A Plant-Produced in vivo deglycosylated full-length Pfs48/45 as a Transmission-Blocking Vaccine Candidate against malaria
Source: Sci Rep. 2019 Jul 8;9:9868. doi: 10.1038/s41598-019-46375-6 (PMC6614448; doi:10.1038/s41598-019-46375-6)
Supplement: Supplementary file 1 — Supplementary Information [file 41598_2019_46375_MOESM1_ESM.pdf]

Supplementary Information

A Plant-Produced *in vivo* deglycosylated full-length Pfs48/45 as a Transmission-Blocking Vaccine Candidate against malaria

Tarlan Mamedov[1,2*], Kader Cicek[1], Kazutoyo Miura[3], Burcu Gulec[1], Ersin Akinci[1] , Gunay Mammadova[1] and Gulnara Hasanova[1]

[1] Akdeniz University, Department of Agricultural Biotechnology, Dumlupınar Boulevard 07058 Campus, Antalya, Turkey

[2] Azerbaijan National Academy of Science, Department of Biology and Medical Science, 24 Istiglaliyyat Street, Baku, Azerbaijan

3 Laboratory of Malaria and Vector Research, National Institute of Allergy and Infectious Diseases, National Institutes of Health, 12735 Twinbrook Parkway, Rockville, MD USA

* Correspondence: tmammedov@gmail.com; Tel.: +90-537-202-4759

Supplementary Table S1: SMFA results[a]

Feed #	Sample name	Mean oocysts	%TRA[b]			
			estimate	95% CI Lo	95% CI Hi	p-value
1	Negative control	18.1				
	dPfs48/45 (E)	0.1	**99**	98	100	0.001
	dPfs48/45 (P)	1.7	**91**	79	96	0.001
	dPfs48/45-10C (E)	4.3	**76**	48	91	0.002
2	Negative control	12.4				
	dPfs48/45-10C (P)	5.8	**53**	1	78	0.050
	g Pfs48/45	14.4	**-16**	-151	45	0.721
	g Pfs48/45-10C	20.9	**-68**	-269	22	0.196
3	Negative control	48.2				
	dPfs48/45 (E)	1.0	**98**	96	99	0.001
	dPfs48/45 (P)	23.2	**52**	-4	78	0.066
	dPfs48/45-10C (E)	37.1	**23**	-68	64	0.460
	dPfs48/45-10C (P)	28.2	**41**	-21	72	0.155
	g Pfs48/45	50.8	**-5**	-129	53	0.858
	g Pfs48/45-10C	63.3	**-31**	-186	39	0.497

[a] Purified IgGs were tested at 0.75 mg/ml with complement, and 20 mosquitoes were dissected per group for tests, and 40 mosquitoes for control.

[b]The best estimates of % inhibition in oocyst density (%TRA), the 95% confidence intervals (95%CI), and p-values from single or multiple feeds were calculated using a zero-inflated negative binomial random effects model (ZINB model) described previously[36].

Supplementary Figure S1. HPLC analysis of plant produced Pfs48/45 (a) and Pfs48/45-10C (b) proteins. All peaks in the chromatogram was detected at 280 nm. The purity was calculated as a percentage of peak area in relation to total area of peaks.

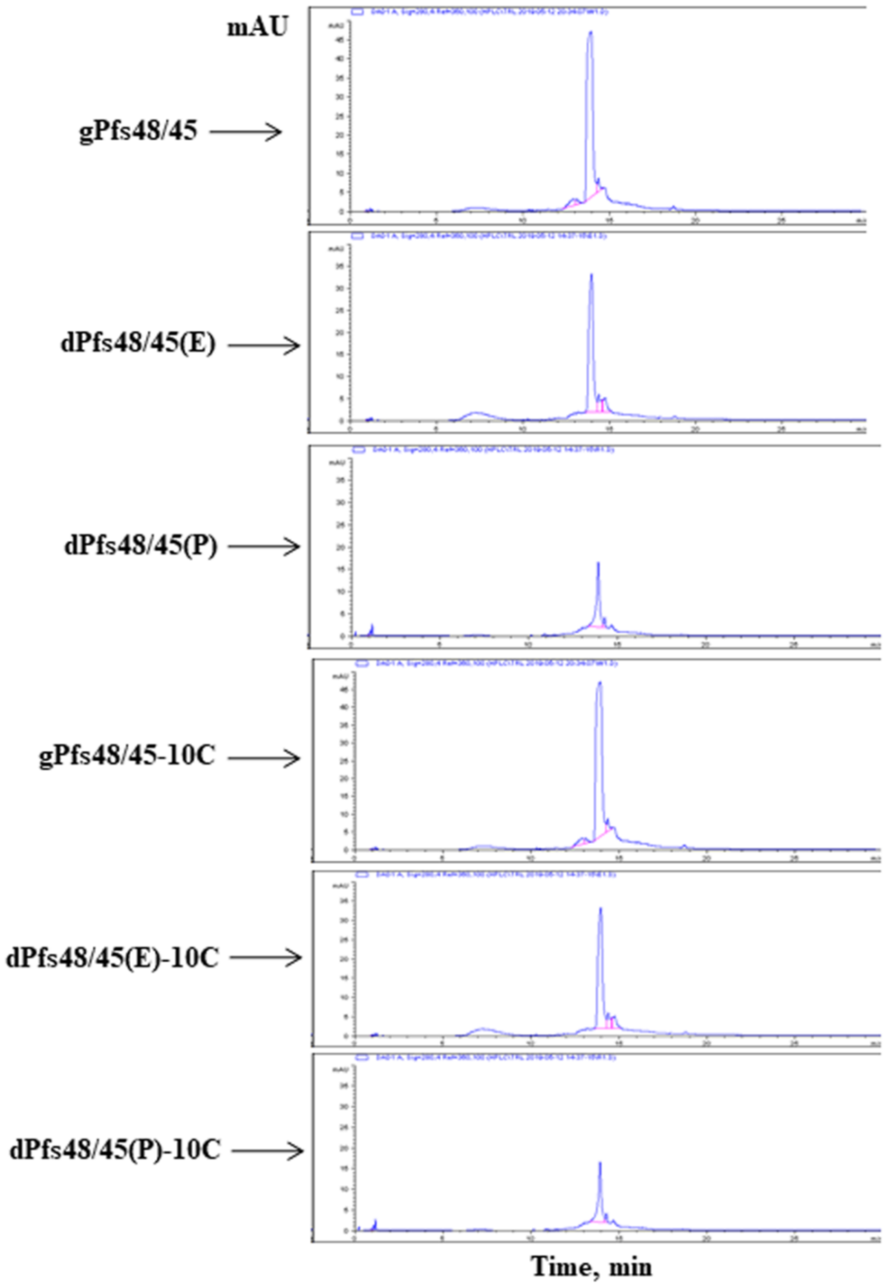

Full-length gels and blots

1. Full-length gels

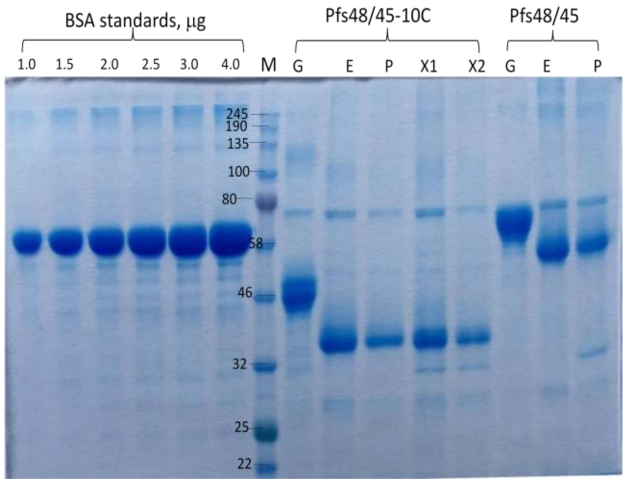

Full-length gel 1. SDS-PAGE analysis of purified, plant produced Pfs48/45 and Pfs48/45-10C variants. Figure 2 (a) indicated as "Pfs48/45" was cropped from this gel (Pfs48/45, Lanes G, E and P along with prestained protein standards, M).

X1, X2: combined last fractions of Pfs48/45-10C-E and Pfs48/45-10C-P, respectively, eluted from anti-FLAG column. M: color prestained protein standard (cat. no. P7712 S New England Biolabs)

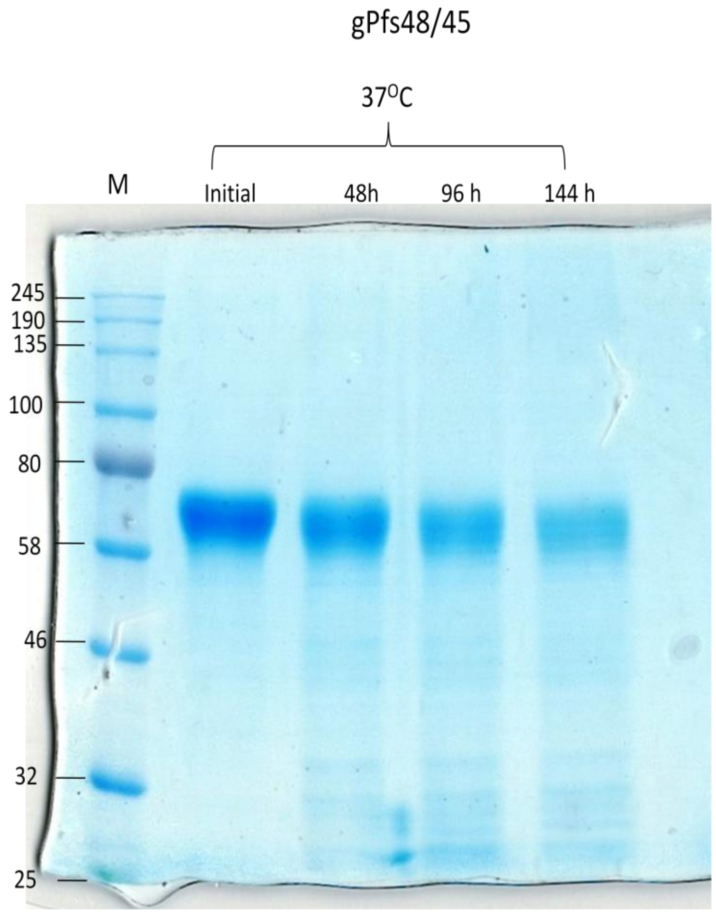

Full-length gel 2. Figure 3 (a), indicated as "gPfs48/45" was cropped from this gel. (in the previous version of the manuscript). Full-length gel 2. was used in this version of the manuscript as suggested by reviewer #3.

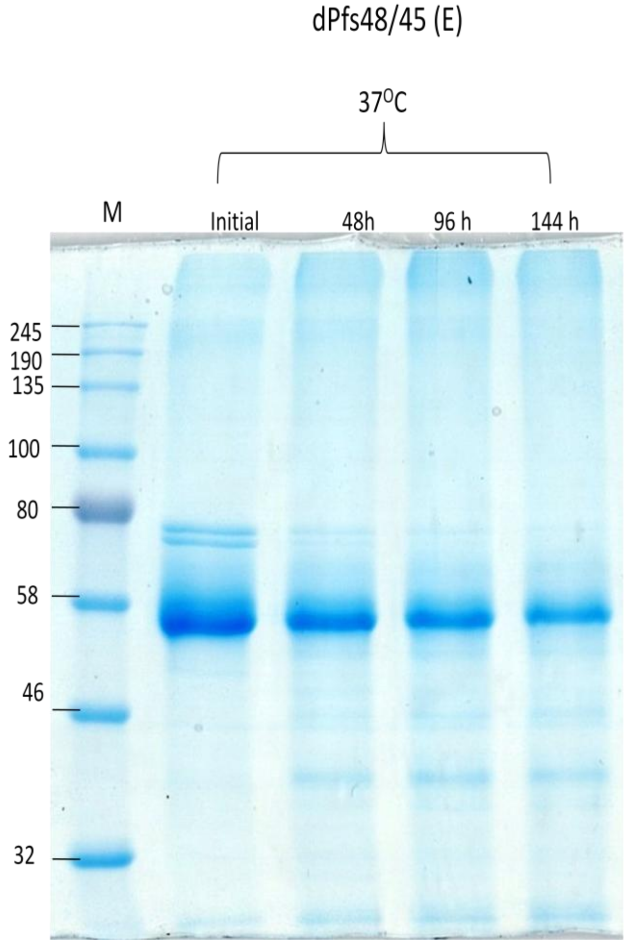

Full-length gel 3. Figure 3 (a), indicated as "dPfs48/45 (E)" was cropped from this gel. (in the previous version of the manuscript). Full-length gel 3. was used in this version of the manuscript as suggested by reviewer #3.

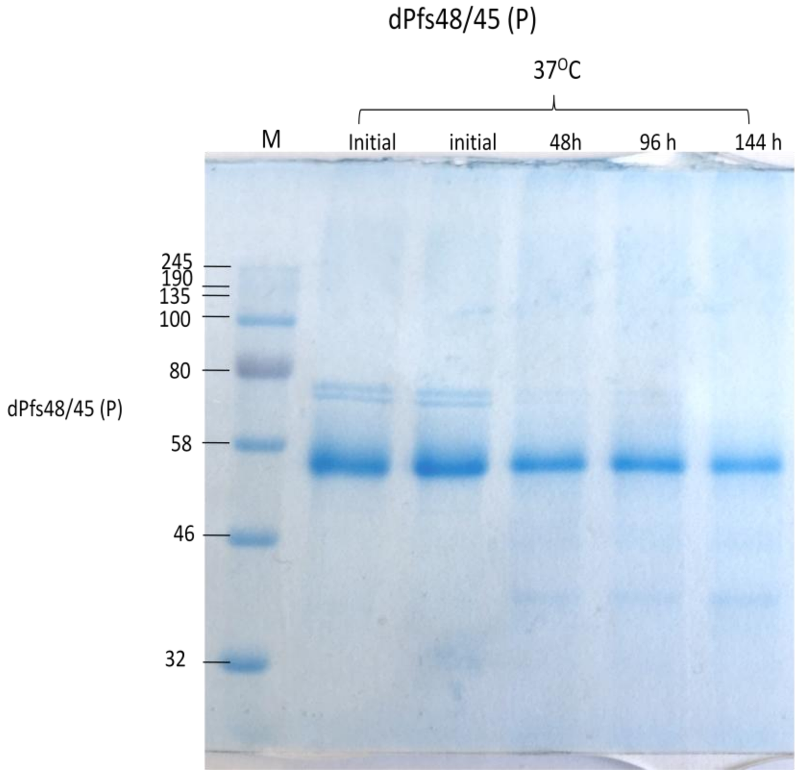

Full-length gel 4. Figure 3 (a), indicated as "dPfs48/45 (P)" was cropped from this gel (in the previous version of the manuscript). Full-length gel 4. was used in this version of the manuscript as suggested by reviewer #3.

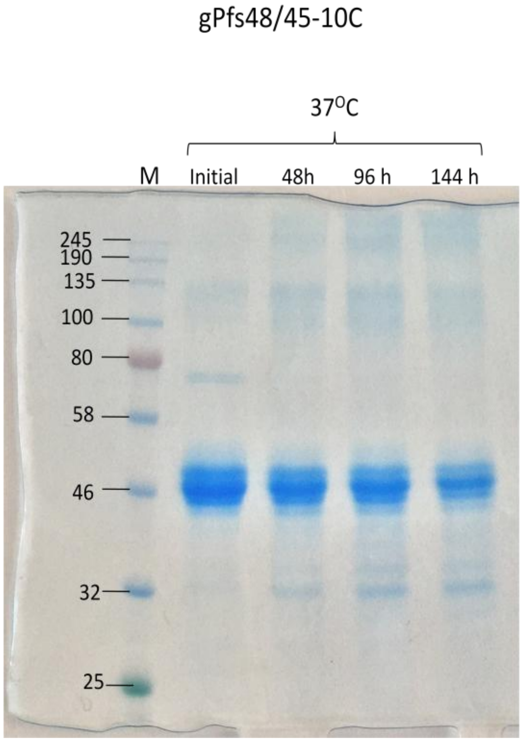

Full-length gel 5. Figure 4 (a), indicated as "gPfs48/45-10C" was cropped from this gel.

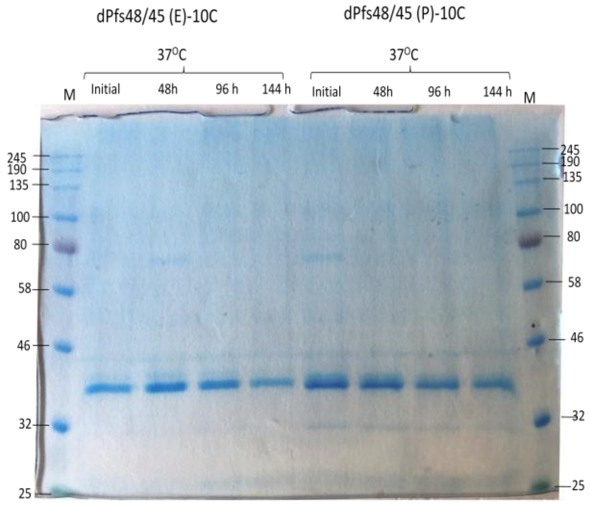

Full-length gel 6. Figure 4 (a), indicated as "dPfs48/45 (P)-10C" and "dPfs48/45 (P)-10C" were cropped from this gel.

__**Full-length blots**__

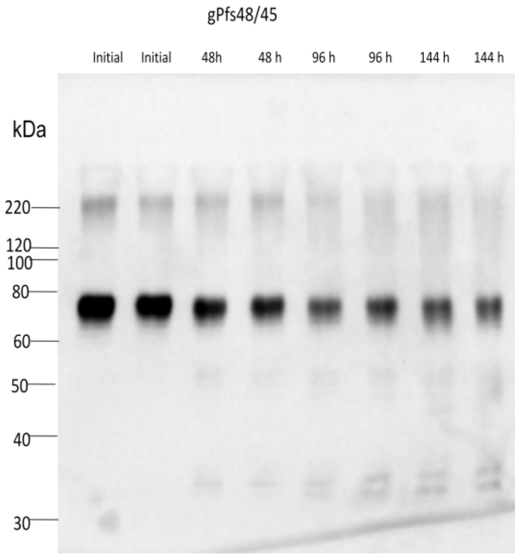

Full-length blot 1. Figure 4 (b), indicated as "gPfs48/45" was cropped from this blot.

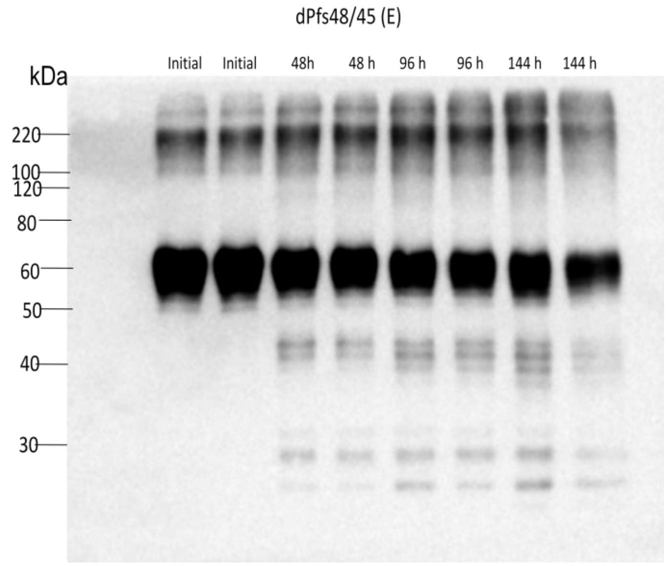

Full-length blot 2. Figure 4 (b), indicated as "dPfs48/45(E)" was cropped from this blot.

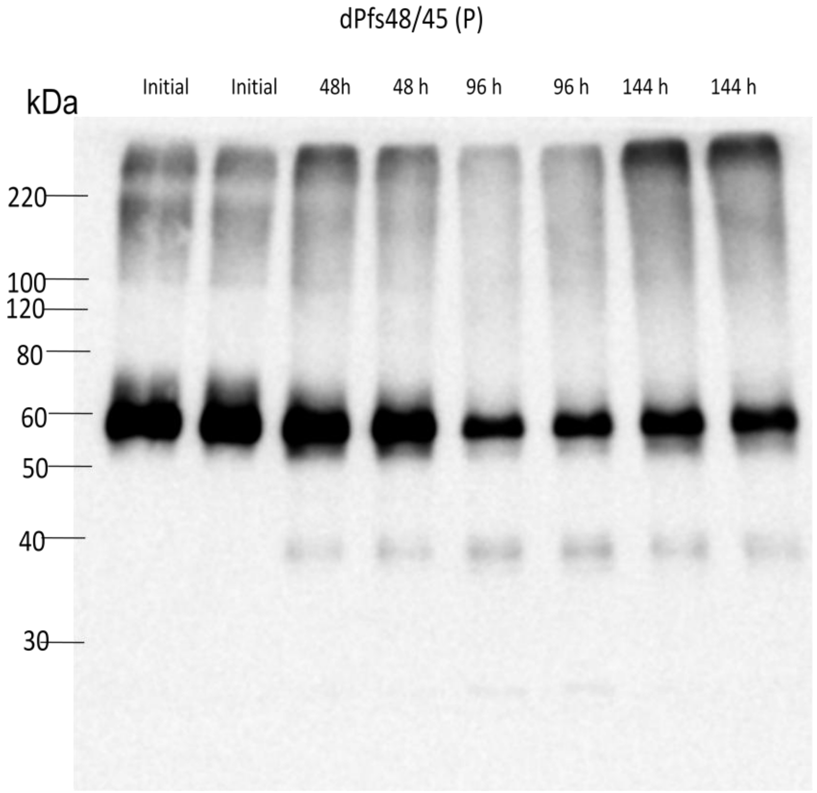

Full-length blot 3. Figure 4 (b), indicated as "dPfs48/45(P)" was cropped from this blot.

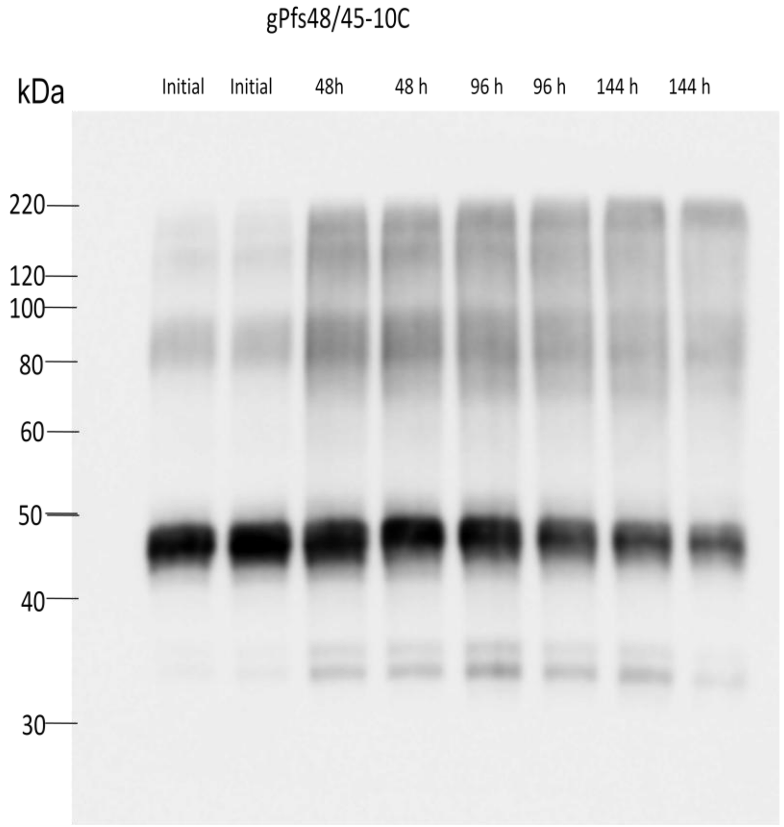

Full-length blot 4. Figure 4 (b), indicated as "gPfs48/45-10C" was cropped from this blot.

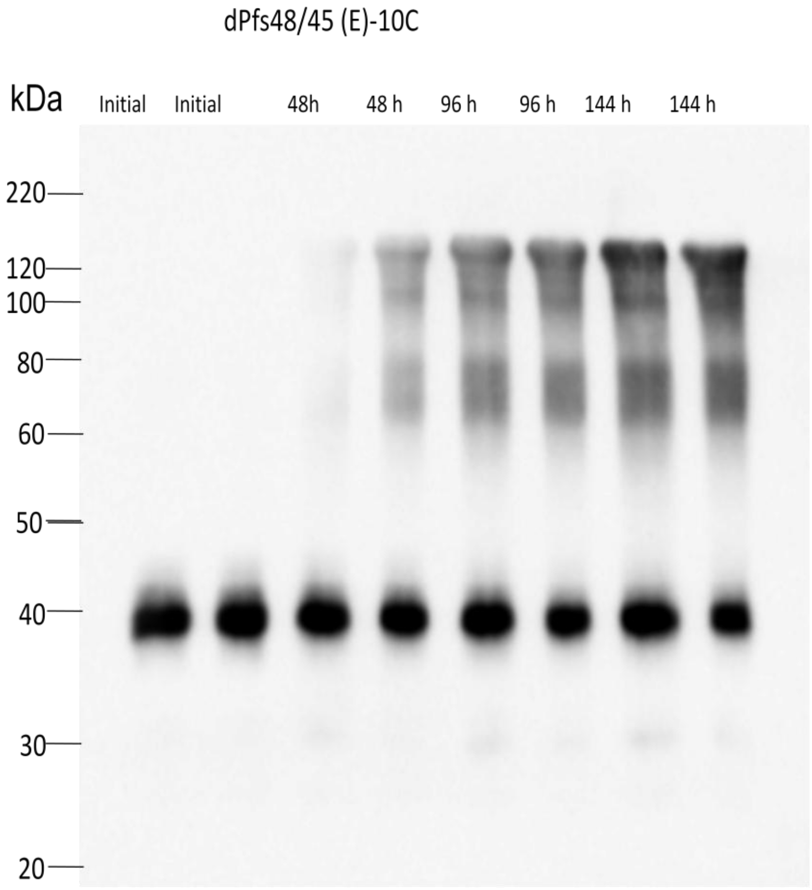

Full-length blot 5. Figure 4 (b), indicated as "dPfs48/45(E)-10C" was cropped from this blot.

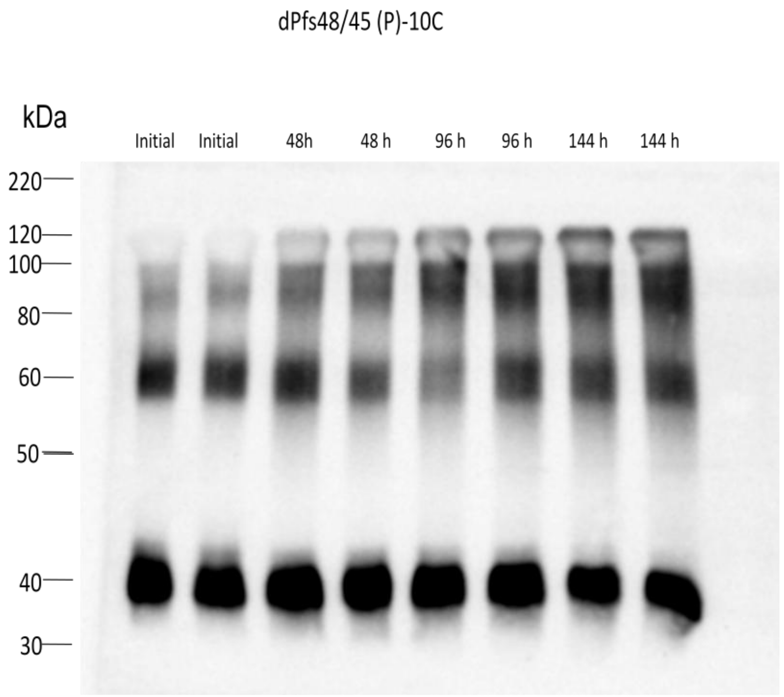

Full-length blot 6. Figure 4 (b), indicated as "dPfs48/45(P)-10C" was cropped from this blot.
